# Defining the larval habitat: abiotic and biotic parameters associated with *Anopheles farauti* productivity

**DOI:** 10.1186/s12936-019-3049-7

**Published:** 2019-12-11

**Authors:** Kimberley McLaughlin, Thomas R. Burkot, Jance Oscar, Nigel W. Beebe, Tanya L. Russell

**Affiliations:** 1 James Cook University, Australian Institute of Tropical Health and Medicine, Cairns, QLD 4870 Australia; 2 Western Province Malaria Control, Gizo, Western Province, Solomon Islands; 3 School of Biological Sciences, University of Queensland, St. Lucia, QLD 4068 Australia; 4 CSIRO, Dutton Park, Brisbane, QLD 4001 Australia

**Keywords:** *Anopheles farauti*, *Anopheles hinesorum*, *Anopheles lungae*, Malaria, Receptivity, Elimination, Density, Wing length, Solomon Islands

## Abstract

**Background:**

In the Solomon Island, the dominant malaria vector, *Anopheles farauti*, is highly anthropophagic and increasingly exophilic and early biting. While long-lasting insecticide-treated nets remain effective against *An. farauti*, supplemental vector control strategies will be needed to achieve malaria elimination. Presently, the only World Health Organization recommended supplemental vector control strategy is larval source management (LSM). Effective targeted larval source management requires understanding the associations between abiotic, chemical and biological parameters of larval habitats with the presence or density of vector larvae.

**Methods:**

Potential and actual *An. farauti* larval habitats were characterized for presence and density of larvae and associated abiotic, chemical and biological parameters.

**Results:**

A third of all sampled potential habitats harboured *An. farauti* larvae with 80% of *An. farauti* positive habitats being in three habitat classifications (swamps/lagoons, transient pools and man-made holes). Large swamps were the most abundant positive habitats surveyed (43% of all *An. farauti* positive habitats). Habitats with *An. farauti* larvae were significantly associated with abiotic (pH, nitrate, ammonia and phosphate concentrations and elevated temperature) and biotic (predators) parameters.

**Conclusion:**

Large swamps and lagoons are the largest and most abundant *An. farauti* habitats in the Solomon Islands. Positive habitats were more frequently associated with the presence of predators (vertebrates and invertebrates) and higher water temperatures. Cohabitation with predators is indicative of a complex habitat ecosystem and raises questions about the potential of biological control as an effective control strategy. Increased presence of *An. farauti* with higher water temperature suggests a potential explanation for the coastal distribution of this species which is not found inland at elevated altitudes where temperatures would be cooler.
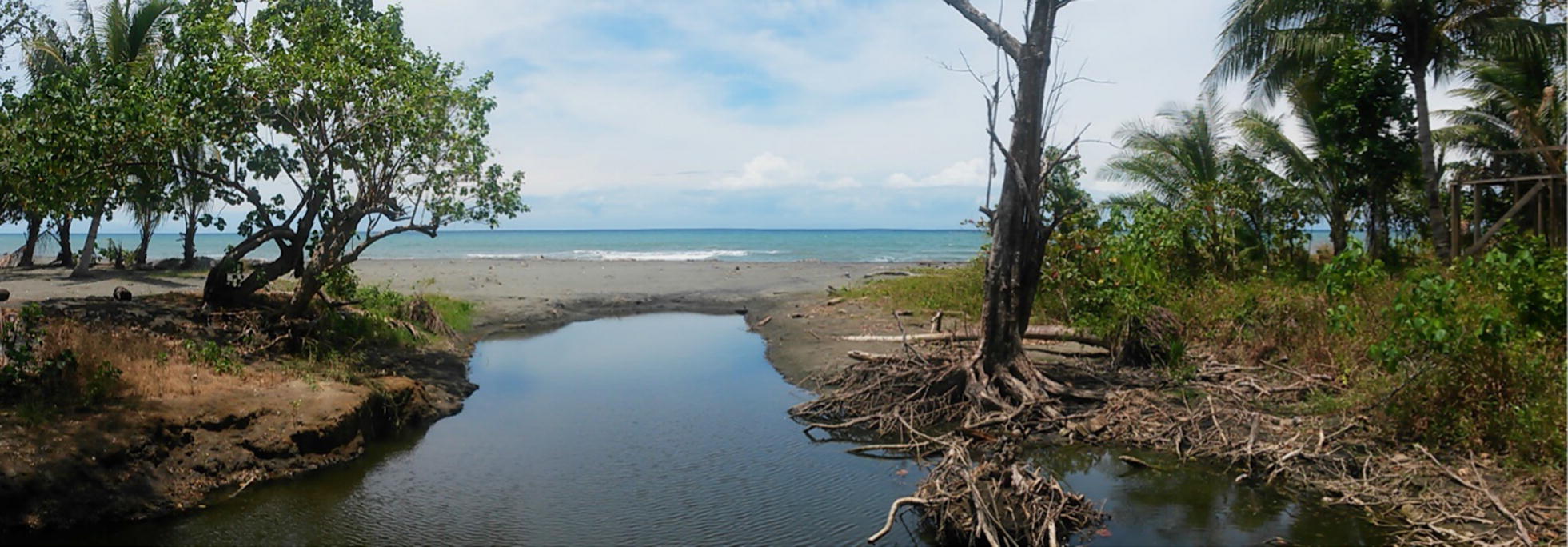

## Background

The geographic distribution of malaria vector species is an important component defining the receptivity of an area for malaria and for stratifying interventions for their control [1]. The World Health Organization (WHO) Global Technical Strategy for Malaria Control and Elimination recommends universal access to vector control with either long-lasting insecticide-treated nets (LLINs) or indoor residual spraying (IRS) to all people at-risk of malaria [2]. However, the recent stabilization in the number of global malaria cases suggests that novel interventions tools will be required to further reduce malaria transmission [3]. At the present time, the only WHO recommended strategy to control vectors outdoors is larval source management (LSM) in areas with seasonal transmission or where the larval habitats are few in number, fixed in location and easily accessible (including urban areas) [2, 4]. Understanding larval habitats is necessary for implementing effective LSM.

Mirroring the global pattern, the Solomon Islands achieved significant reductions in malaria cases following universal distribution of LLINs, but the number of cases has increased since 2015 to a prevalence of 80 cases/1000 population in 2017 (Solomon Island Annual Malaria Programme Report, 2017). Six members of the *Anopheles punctulatus* group are found in the Solomon Islands [5]. *Anopheles farauti* is the dominant malaria vector [6]. *Anopheles punctulatus*, once found across the Solomon Islands, is now confined to Guadalcanal and Malaita Provinces as a consequence of the IRS campaigns with DDT in the 1970s [7]. *Anopheles koliensis*, a major vector in Papua New Guinea [8], may have been eliminated from the Solomon Islands as a consequence of IRS with DDT [9], as this species has not been found in in the Solomon Islands since 1987 [7]. While *Anopheles hinesorum* is a malaria vector in Papua New Guinea [10], only a single sporozoite positive individual was found in Western Province, Solomon Islands and this species is unlikely to maintain malaria transmission due to its predominantly zoophagic biting habit in the Solomon Islands [6]. The vector status of *Anopheles rennellensis* is unknown, and *Anopheles irenicus* has not been collected using human landing catches and so is not regarded as a malaria vector [8]. Hence, malaria vector control in the Solomon Islands focuses on *An. farauti*.

Recent surveys in Central and Western Provinces identified larval habitats of *An. farauti*, *An. hinesorum*, *Anopheles lungae*, *Anopheles solomonis* and *Anopheles nataliae*, the latter three species belonging to the *An. lungae* complex [11, 12]. Larvae of *An. lungae, An. solomonis* and *An. nataliae* were most frequently found in riverine habitats, with *An. lungae* also found in swamps and lagoons in Central and Western Provinces [11]. While *An. farauti* and *An. hinesorum* were found in a range of habitat types, the most frequently utilized were coastal lagoons and swamps, hypothesized to be responsible for producing most adults [11], as the highest populations of *An. farauti* are found in villages near large brackish water lagoons and freshwater swamps [6, 13, 14].

Characterization of *An. farauti* larval habitats to date has been limited. *Anopheles farauti* has only been found predominantly near the coast [10]. On Guadalcanal, *An. farauti* larvae are associated with emergent plants and filamentous algae with highest larval densities found closest to the mouths of saline lagoons [15]. This study aimed to determine abiotic and biotic parameters associated with prevalence and productivity of anophelines in the Western and Central Provinces of the Solomon Islands. Understanding the characteristics associated with *An. farauti* larvae may enable rapid identification of productive habitats for targeted larval control.

## Methods

### Study period and sites

**Fig. 1:**
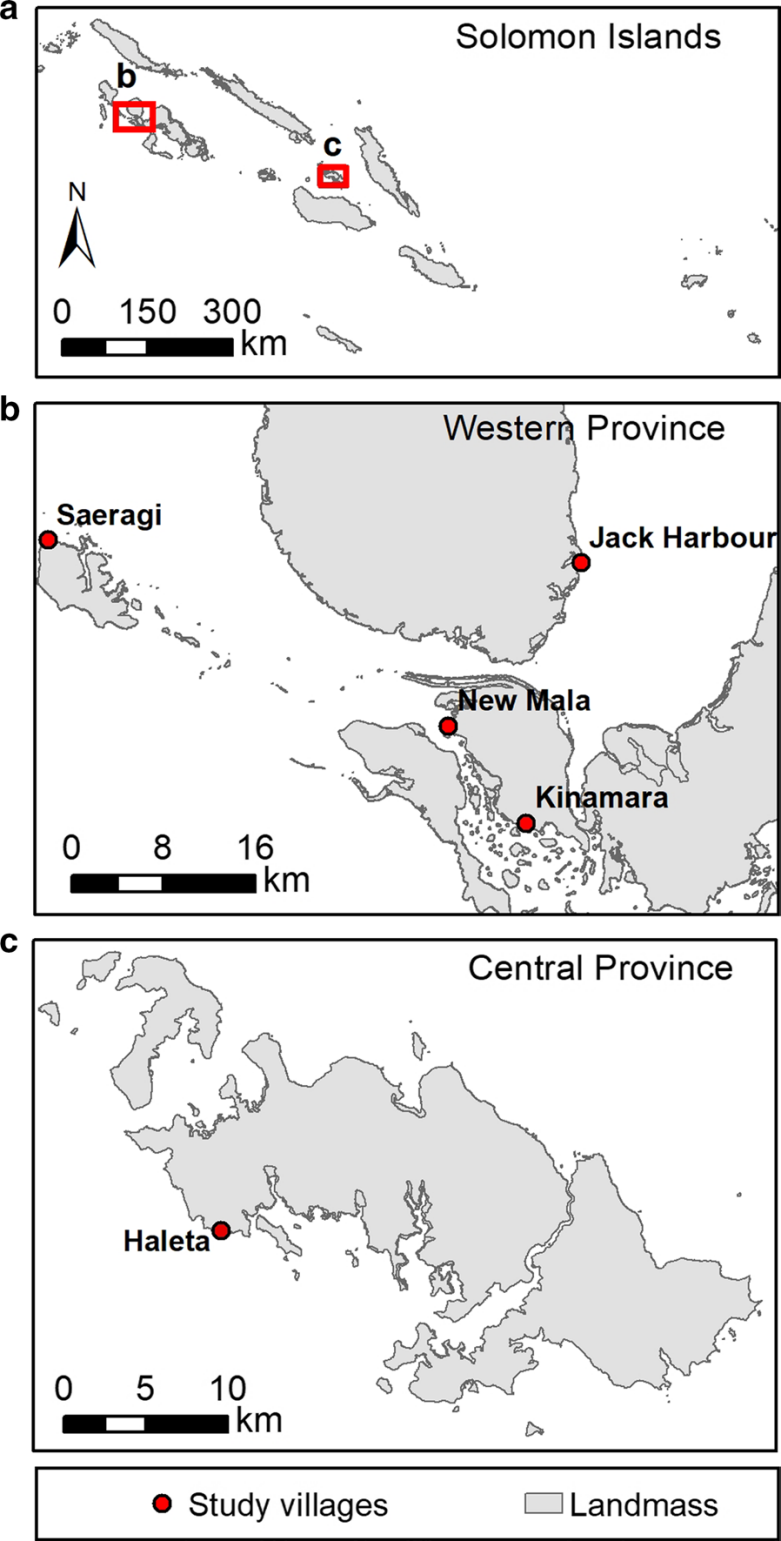
Map of (**a)** the Solomon Islands showing locations of (**b)** the 4 study villages in Western Province (8°0′ S, 157°0′ E) and (**c)** Haleta village in Central Province (9°0′ S, 159°45′ E)

Larval surveys were conducted during January and August 2016 near villages in Western Province (Jack Harbour, Saeragi, Kinamara, and New Mala; − 8.0° S, 157.0° E) and Central Province (Haleta; − 9°5′ 56″ S, 160°6′ 56″ E) of the Solomon Island (Fig. 1) [6]. The villages are on volcanic, rain-forested mountainous islands. The climate of the region is hot and wet with annual rainfall of 3725 mm and 2837 mm for New Georgia Island, Western Province and Central Province, respectively, from 1999 to 2010 (Solomon Islands Meteorology Department for Munda Airport, Western Province, and Henderson Airport for Central Province, unpublished data). The mean daily minimum and maximum temperatures of both provinces were 24 °C and 30 °C, respectively, with an overall mean of 26 °C.

### Habitat characterization

The study villages and surrounding areas were extensively searched for potential larval habitats. Potential larval habitats were sampled for mosquito immature stages using 250 ml dippers, with 10 samples (dips) taken at each station. Habitats with water but without larvae present were recorded as negative habitats, areas with water and larvae were recorded as “positive habitats” and areas previously with water but now dry recorded as potential habitats. Habitats with perimeters larger than 10 m were sampled at multiple stations between 5 and 10 m apart. The geoposition of sampling stations were recorded (Juno Trimble 3D). Larvae captured were counted by instars and stored in 70% ethanol for subsequent identification by PCR using the internal transcribed spacer region II of ribosomal DNA (ITS2) [16].

Environmental parameters included abiotic and biotic categories that were further defined by sub-categories (classifications) (an additional table provides further detail, see Additional file 1: Table S1). Habitat classifications were transient pools, lagoon or swamp, drains, man-made holes, water storage containers, riverine habitats, ponds, and rock pools.

Abiotic characteristics included the substrate (habitat floor), water depth, bank slope, size (as defined by perimeter circumference), light intensity, debris present, chemical concentrations and temperature. Substrate classifications were rock, gravel, sand and silt [17]. Water depth was measured approximately 30 cm from the habitat edge (or centrally for small habitats) and analysed in 5 cm increments. Bank slope was recorded as gentle (0–19° angle), moderate (20–49° angle) and steep (50–90° angle). Habitat perimeters were classified as small (< 10 m), medium (10–100 m), and large (> 100 m). Light intensity was classified by the amount of sunlight illuminating the habitat (i.e., none, partial sun, or full sun). Debris classes of natural or man-made materials floating or immersed in the habitat were none, dead plant materials, man-made materials, biological surface film (scum), and floating pumice. Temperature, salinity and pH were quantified with a Cyberscan series 600 PCD 650 meter. Chemicals measured were sodium chloride (salinity), nitrate (mg/L), ammonia (mg/L) and phosphate (mg/L) Nitrate, ammonia and phosphate concentrations were measured semi-quantitatively using Quantofix test strips (Macherey–Nagel; Duren, Germany).

Biotic parameters were classified by canopy (vegetation above habitats), vegetation (living within the aquatic habitat), and predators. Canopy classifications were none, shrub, and tree. Vegetation classifications were none, trees, bushes, algae, floating vegetation, and emergent. Predator classifications were fish, tadpoles, dragonfly nymphs, water striders, and others.

### Statistical analysis

The influence of environmental parameters on the presence and density of anopheline larvae was analysed using Generalized Linear Mixed Models (GLMM) with a unique identifier for habitat as a random variable to account for repeated sampling. The data was analysed with two different distributions: (1) binary data (presence and absence) was fitted to a binomial distribution, and (2) count data was fitted to a negative binomial distribution as count data was not normally distributed. Larval collection stations with zero larvae counts were excluded from density analyses. All analyses were conducted using the *R* package V3.6.0 [18].

## Results

### Larval distribution

Anopheline larvae were sampled from 67 larval habitats, 41 in Western Province (Jack Harbour: n = 13; Kinamara: n = 6; New Mala: n = 10; and Saeragi: n = 12) and 26 in Haleta village in Central Province. The number of sample stations per habitat ranged from 1 to 24, depending on habitat size. Overall, 324 anopheline larvae were identified to species by PCR. *Anopheles farauti* was the most abundant and widespread species, being found in all surveyed villages. *Anopheles hinesorum* larvae were only collected in Kinamara village, where this species made up 79% of identified specimens.

*Anopheles farauti* immatures were found in a wide range of habitat classifications: coastal lagoons and swamps, transient pools, man-hade holes, riverine habitats, drains and ponds. The most commonly occupied habitats were lagoons and swamps, transient pools and man-made holes (Table 1).

**Table 1:** Larval habitats of *An. farauti* s.s. in Central and Western Provinces, Solomon Islands

Habitat classification	Number habitats	Number positive	Habitat classification positive (%)
Lagoon or swamp	42	12	29
Transient pools	34	8	24
Man-made holes	20	8	40
Rivers	6	4	67
Drains	2	2	100
Pond	1	1	100
Water storage containers	2	0	0
Grand total	107	35	33

### Larval prevalence by abiotic and biotic parameters

**Fig. 2:**
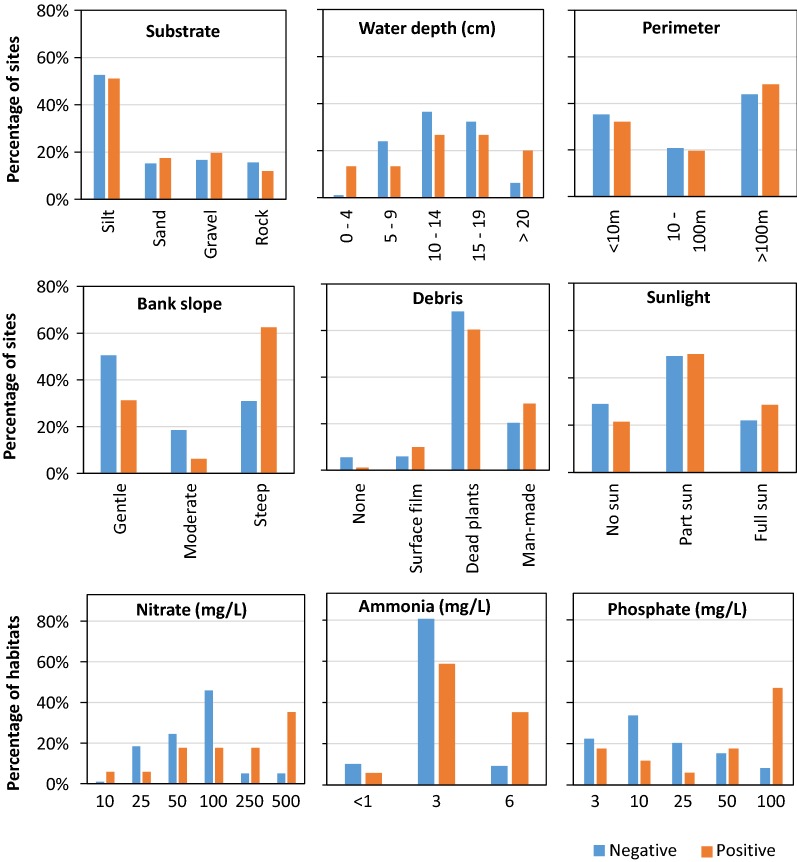
Associations of abiotic parameters analysed categorically against the presence of *An. farauti* larvae in aquatic habitats

**Fig. 3:**
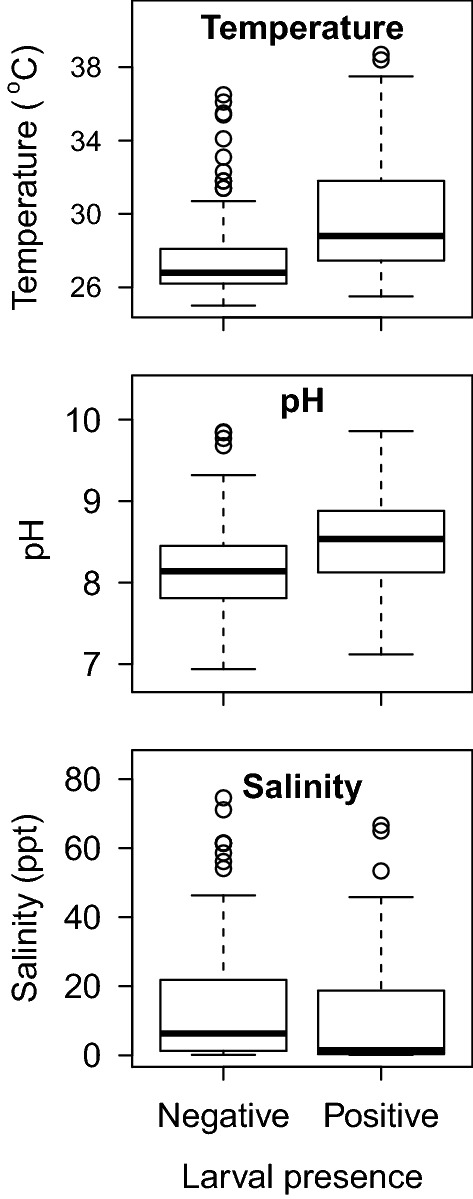
Associations between abiotic parameters analysed continuously against the presence of *An. farauti* larvae in larval habitats

**Fig. 4:**
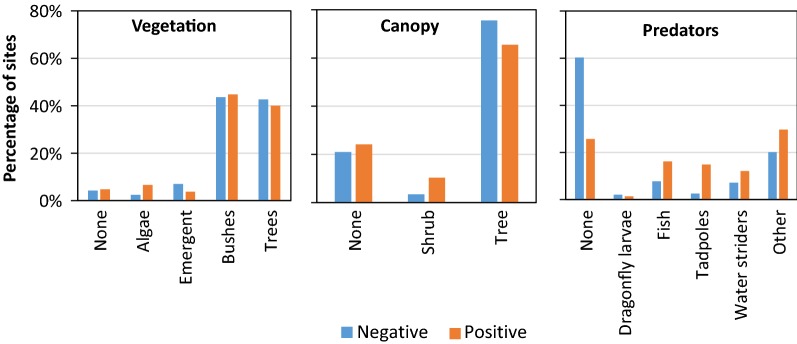
Associations between biotic parameters and the presence of *An. farauti* larvae in aquatic habitats

Associations of abiotic and biotic parameters with larvae presence are shown in Figs. 2, 3 and 4. Six larval habitat parameters were significantly and positively associated with *An. farauti* presence (Table 2): predators (*P* = 0.018), temperature (*P* < 0.001), pH (*P* = 0.008), nitrate (*P* = 0.005), ammonia (*P* = 0.022) and phosphate (*P* = 0.003). The mean temperature of larval positive habitats (29.8 °C) was 2.3 °C greater than the average temperature of negative habitats. Positive larval habitats had a mean pH of 8.5, whereas negative sites had a mean pH of 8.1. *Anopheles farauti* larvae were found significantly more frequently in habitats with concentrations of nitrate > 250 mg/L, at ammonia concentrations of 6 mg/L or phosphate concentrations of 100 mg/L. Parameters not associated with larvae presence were substrate, water depth, habitat size, bank slope, canopy type, sunlight, vegetation, debris and salinity.

**Table 2:** Association of abiotic and biotic parameters with the presence and density of *An. farauti* larvae in Central and Western Provinces, Solomon Islands

Parameter	Binary (presence/absence) model			Negative binomial (density) model		
	*β*	*SE*	*P* value	*β*	*SE*	*P* value
Substrate	− 0.016	0.1388	0.907	− 0.038	0.0731	0.600
Water depth	0.166	0.2015	0.408	0.049	0.0379	0.196
Perimeter	0.154	0.2690	0.564	− 0.260	0.1966	0.185
Bank slope	2.149	1.2800	0.093˙	0.093	0.3071	0.760
Canopy type	− 0.058	0.2364	0.805	− 0.112	0.1533	0.466
Sunlight	0.137	0.2144	0.522	0.159	0.1841	0.385
Vegetation	− 0.053	0.0991	0.586	− 0.044	0.0551	0.416
Debris	− 0.200	0.2612	0.443	0.009	0.1502	0.950
Predators	0.365	0.1551	0.018*	0.001	0.0897	0.985
Temperature	0.293	0.0739	< 0.001*	0.116	0.0398	0.003*
pH	0.863	0.3242	0.008*	0.457	0.2593	0.077˙
Salinity	0.061	0.1799	0.733	0.092	0.1592	0.563
Nitrate	0.006	0.0023	0.005*	− 0.154	0.3041	0.611
Ammonia	0.752	0.3294	0.022*	0.221	0.1428	0.120
Phosphate	0.028	0.0095	0.003*	− 0.418	0.2770	0.131

Data were compared with GLMMs with the habitat identifier as a random variable to account for repeated sampling. The data was analysed with two different distributions: (1) binary data (presence or absence) was fitted to a binomial distribution, and (2) count data were fitted to a negative binomial distribution. For the density analysis, all sampling sites with zero counts of larvae were excluded

### Larval density and abiotic, chemical and biological parameters

Associations of abiotic and biotic parameters with larval density (the total number of larvae per 10 dips) are summarized in additional figures (see Additional file 2: Figures S1, S2, S3). Temperature was significantly and positively associated with *An. farauti* density (*P* = 0.003) (Table 2).

## Discussion

Larval habitats in the Solomon Islands were examined for associations between abiotic and biotic parameters and the prevalence or density of *An. farauti* larvae. This research was conducted in response to previous studies that had highlighted that the distribution of *An. farauti* larvae was not uniform [11, 15]. During the current survey, only a third of potential habitats contained *An. farauti* during two surveys, and markers that might be used for targeting habitats with immature stages were sought. Here, the most commonly occupied habitats were lagoons and swamps, transient pools and man-made holes, which was similar to previous surveys in the region [11].

Quantitative larval surveys are fraught with challenges, particularly in large habitats as larvae are not uniformly distributed and thus requires sampling multiple sites within a habitat to establish with some certainty if and where larvae are present, particularly for anophelines which are frequently found in low densities. Distinguishing high from low productivity of large habitats is challenging as the emergence rates of adults will be a function of mature larval density, rate of development (a function of multiple factors including nutrient sources and temperature), the size of the habitat and the moderating impact of predators located in the habitat. A final challenge is that larval counts in a dipper cannot be related to either density of larvae per habitat area or numbers of adults [19, 20].

Hence, analyses of environmental habitat parameters for productivity are better served by looking for associations with the presence of larvae rather than the density of larvae. Significant associations of *An. farauti* larval presence and nitrate, ammonia and phosphate concentrations, pH and temperature were found. It is generally accepted that the development of anopheline larvae is strongly influenced by the abiotic and biotic factors of the aquatic habitats. For example, *Anopheles gambiae* larval densities depend on variables such as nitrogen, temperature, pH, dissolved oxygen and salinity [21–25]. Here similar parameters also influence the presence of *An. farauti* larvae. This supports the literature demonstrating the association of *An. farauti* with salinity [15, 26, 27]. The increased association of habitats with elevated temperatures with *An. farauti* presence is consistent with the distribution records for this species and may be a factor in explaining its predominantly coastal distribution.

Interestingly, *An. farauti* larvae were significantly more often found associated with the presence of predators suggesting co-habitation of more complex aquatic ecosystems (and raising concerns about the potential impact of biological control). Similar observations were made for *Anopheles albimanus* in the Pacific region of Colombia, where larval density was highest in sites with abundant and varied aquatic fauna, including different predators [28]. Similarly, there are examples where *An. gambiae* are more abundant in habitats containing tadpoles [24]. This provides further evidence to indicate that the relationship between the biotic and abiotic parameters and oviposition choice and larval development is complex.

## Conclusions

Associations between abiotic and biotic parameters and larval presence suggests that assaying habitats for these parameters might be a more efficient means to select targets for larval source management compared to traditional larval surveys. Overall the findings in this study support the idea that larval control is a feasible option for vector control that could complement the wide-scale use of LLINs and IRS in the region. Especially, as the primary larval habitat of *An. farauti* in the Solomon Islands were “few (in number), fixed (permanent swamps) and findable (located close to villages)” [29]. However, the entomological and epidemiological evidence to support the implementation of LSM is required.

## Supplementary information

**Additional file 1: Table S1.** The definitions and classifications for environmental parameters of larval habitats.

**Additional file 2: Figure S1.** Comparison of the influence of abiotic parameters that were analysed categorically on the density of *An. farauti* larvae in aquatic habitats. **Figure S2.** Comparison of the influence of abiotic parameters that were analysed continuously on the density of *An. farauti* larvae in aquatic habitats. **Figure S3.** Comparison of the influence of biotic parameters on the density of *An. farauti* larvae in aquatic habitats.

## Data Availability

The datasets supporting this article are available from the JCU Tropical Data Hub repository: http://dx.doi.org/10.25903/5d3fd8a0a957c.

## Abbreviations

IRS: indoor residual spraying
LLIN: long-lasting insecticide treated nets
LSM: larval source management
WHO: World Health Organization
