# Supplementary material for: Defining the larval habitat: abiotic and biotic parameters associated with Anopheles farauti productivity
Source: Malar J. 2019 Dec 11;18:416. doi: 10.1186/s12936-019-3049-7 (PMC6907239; doi:10.1186/s12936-019-3049-7)
Supplement: Supplementary file 1 — Additional file 1: Table S1. The definitions and classifications for environmental parameters of larval habitats. [file 12936_2019_3049_MOESM1_ESM.pdf]

Additional file 1

Table S1. The definitions and classifications for environmental parameters of larval habitats.

Environmental parameter	Classifications	Definitions
Habitat	*Broad classification of the form of each larval habitat (single choice)*	
	Transient pools	Temporary pool of water formed in surface depressions.
	Lagoon or swamp	Low area filled with stagnant water, could be freshwater or a brackish water marsh. Note that this includes blocked stream mouths, were a body of ground water forms when the flow is prevented from discharging by an obstruction (ie., sand bar or similar)
	Drains	A narrow channel parallel to a road or field capable of holding or carrying away water
	Man-made holes	Artificial body of water created by humans, this includes construction pits, concrete ponds, tyre tracks and water wells
	Water storage containers	Man-made tanks/barrels for holding water
	Riverine habitat	A narrow natural flowing water body
	Pond	Permanent (or semi-permanent) natural body of still ground freshwater
	Rock pool	A pool of water that collects in a depression in a rock
Substrate	*Description of the base of the larval habitat (multiple choice) [17]*	
	Rocks	Stones >2 cm in diameter
	Gravel	Stones from 4 mm to 2 cm in diameter
	Sand	Stones from 0.05 and 4 mm in diameter
	Silt	Fine Particles of sand, clay, or other materials <0.05 mm in diameter deposited as a sediment
Water depth	*Distance from the bottom to the surface of an aquatic habitat measured approximately 30 cm from the perimeter of the habitat (single choice)*	
	0 – 4 cm	Water is less than and including 4 cm deep
	5 – 9 cm	Water is between 5 and 9 cm deep
	10 – 14 cm	Water is between 10 and 14 cm deep
	15 – 19 cm	Water is between 15 and 19 cm deep

**Environmental parameter**	**Classifications**	**Definitions**
	>20 cm	Water is more than 20 cm deep
Perimeter	*Perimeter of the habitat, described by its size (single choice)*	
	Small	Habitat with a circumference of less than 10 m
	Medium	Habitat with a circumference between 10 and 100 m
	Large	Habitat with a circumference over 100 m
Bank slope	*The gradient of the land bank at the point where it meets the water surface (single choice) [30] (Figure A1)*	
	Gentle	When the angle of ground forming the sides of a larval habitat ranges from 0 – 19°
	Moderate	When the angle of ground forming the sides of a larval habitat ranges from 20 – 49°
	Steep	When the angle of ground forming the sides of a larval habitat ranges from 50 – 90°
Canopy	*Vegetation coverage above the sampling site (single choice)*	
	None	Absence of any vegetation shading a larval habitat
	Bush	Small- to medium-sized woody plant overhanging the larval habitat
	Tree	Woody plant more that 6 m tall overhanging the larval habitat
Sunlight	*Amount of natural illumination of the larval habitat during the day (single choice)*	
	No sun	Absence of direct solar light
	Partial sun	Variable solar illumination during daytime due to partial obstruction by vegetation
	Full sun	Condition whereby the habitat receives complete solar illumination throughout daytime
Vegetation	*Types of plants living in the sample site (multiple choice)*	
	None	Absence of plants
	Trees	Woody vegetation >6 m tall
	Bushes	Woody vegetation <6m tall
	Algae	Any of a large, diverse group of photosynthetic eukaryotic organisms
	Floating	Plant matter suspended on the habitat surface

**Environmental parameter**	**Classifications**	**Definitions**
	Emergent	Woody or non-woody plants rooted in the bottom of the larval habitat that rise above the surface of the larval habitat
Debris	*The remains of vegetation (non-living) and man-made materials (paper, plastic, metal) in or on the larval habitat (multiple choice)*	
	None	Absence of natural or man-made materials
	Dead plant material	Non-living vegetative material present
	Man-made material	Plastic, metal or paper or other non-natural materials processed by humans
	Scum	Surface film composed of dirt, organic materials or froth (including biofilms) on the larval habitat
	Pumice	Buoyant rocks/gravel present on the habitat surface
Predators	*Animals that naturally prey on immature mosquitoes (multiple choice)*	
	Fish	Gill-bearing aquatic animals lacking limbs with digits
	Tadpoles	Immature amphibians
	Dragonfly nymphs	Nymphs of the order Odonata, family Anisoptera
	Water striders	Insects of the family Gerridae
	Other	Carnivorous animals not in the above classifications
