# Supplementary material for: Defining the larval habitat: abiotic and biotic parameters associated with Anopheles farauti productivity
Source: Malar J. 2019 Dec 11;18:416. doi: 10.1186/s12936-019-3049-7 (PMC6907239; doi:10.1186/s12936-019-3049-7)

Additional file 2

Figure S1: Comparison of the influence of abiotic parameters that were analysed categorically on the density of *An. farauti* larvae in aquatic habitats.

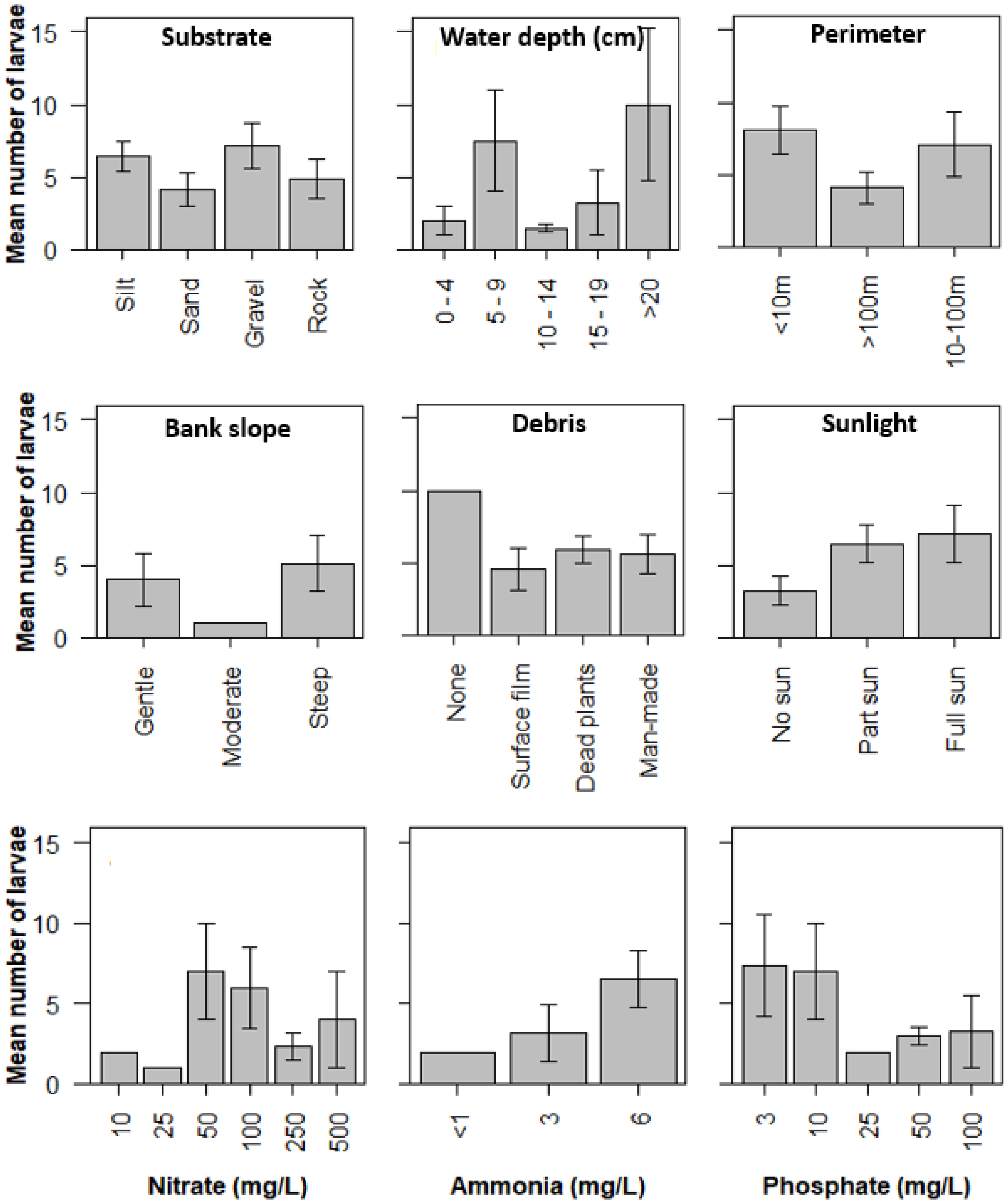

Figure S2. Comparison of the influence of abiotic parameters that were analysed continuously on the density of *An. farauti* larvae in aquatic habitats.

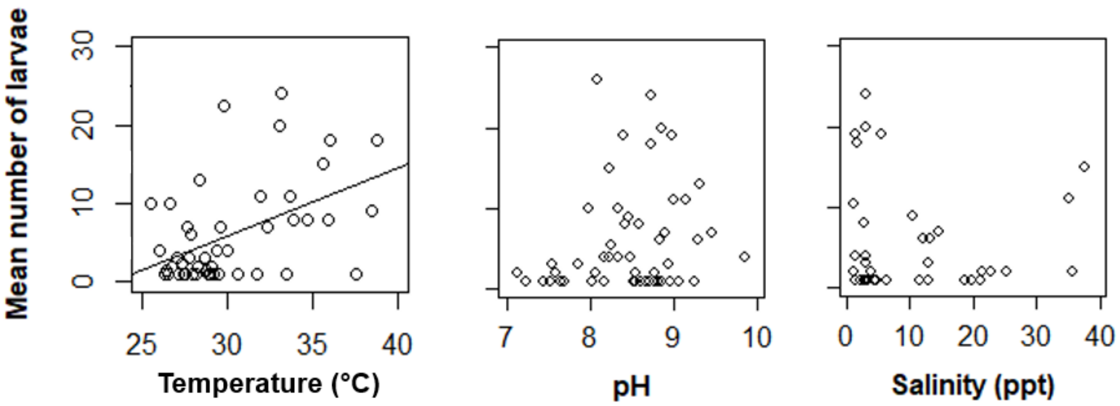

Figure S3. Comparison of the influence of biotic parameters on the density of *An. farauti* larvae in aquatic habitats.

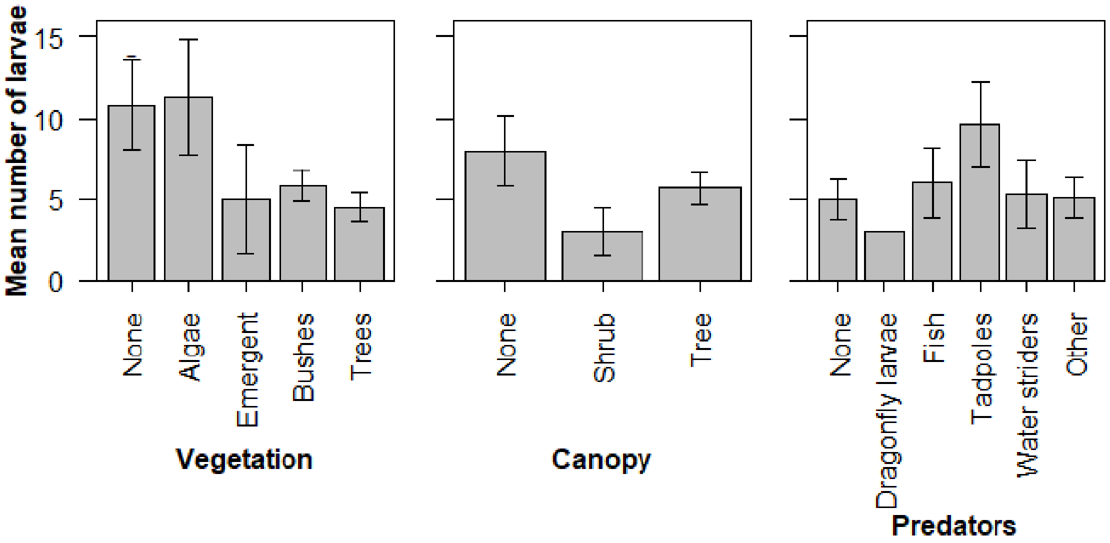

Supplement: Supplementary file 2 — Additional file 2: Figure S1. Comparison of the influence of abiotic parameters that were analysed categorically on the density of An. farauti larvae in aquatic habitats. Figure S2. Comparison of the influence of abiotic parameters that were analysed continuously on the density of An. farauti larvae in aquatic habitats. Figure S3. Comparison of the influence of biotic parameters on the density of An. farauti larvae in aquatic habitats. [file 12936_2019_3049_MOESM2_ESM.pdf]
